# Long-term survival in a patient with advanced non-smoking lung adenocarcinoma and no EGFR mutation after comprehensive therapy with EGFR-TKI-based therapy: A case report

**DOI:** 10.1097/MD.0000000000028583

**Published:** 2022-01-14

**Authors:** Qianyu Zhang, Meiling Liu, Yunchao Zhong, Kang Zhang

**Affiliations:** a Department of Medical Oncology, The First Affiliated Hospital of Guangxi Medical University, Nanning, Guangxi, China; b Department of Medical Oncology, The People's Hospital of Wuzhou, Wuzhou, Guangxi, China.

**Keywords:** epidermal growth factor receptor-tyrosine kinase inhibitor-based therapy, long term survival, no epidermal growth factor receptor gene mutation, patient with advanced lung adenocarcinoma

## Abstract

**Rationale:**

This is the first known report to describe a case of advanced non-smoking lung adenocarcinoma with no epidermal growth factor receptor mutation after comprehensive epidermal growth factor receptor-tyrosine kinase inhibitor-based therapy that survived for nearly 12 years.

**Patient concerns:**

A 48-year-old Chinese woman that came to our hospital with a left supraclavicular mass.

**Diagnosis:**

The final diagnosis was left lung adenocarcinoma with left supraclavicular lymph node metastasis, cT1N3M0 IIIB (International association for the Study of lung cancer 6th version).

**Interventions:**

After 4 months of 4 cycles of chemotherapy, gefitinib was administered alone for 5.5 years. Local radiotherapy (40GY/20F) was administered for 1 month, and osimertinib alone was followed for 4.5 years. A combination of pemetrexed with oxaliplatin and hyperthermia at the same time was administered for 4 cycles. Toripalimab and anlotinib were administered for 3 months. Since then, the patient had been taking a double dose of icotinib herself.

**Outcomes:**

The patient has survived for nearly 12 years since diagnosis of lung cancer.

**Lessons:**

Our case is of significant importance to clinicians involved in the treatment of patients with advanced nonsmoking lung adenocarcinoma and no epidermal growth factor receptor mutations.

## 1 Introduction

Lung cancer has become a common malignant tumor in various countries around the world. The prognosis of patients with advanced non-small cell lung cancer (NSCLC) is poor, with a 1-year survival rate after chemotherapy of only 29%.^[1]^ Epidermal growth factor receptor (EGFR) mutations are important cancer-driving factors. The EGFR mutation rate in female non-smokers with lung adenocarcinoma is approximately 50%. However, the development of epidermal growth factor receptor tyrosine kinase inhibitors (EGFR-TKIs) has dramatically improved the prognosis of certain patients. In the last 10 years, first-generation EGFR-TKIs, such as gefitinib, erlotinib, and icotinib, have become indispensable treatments for EGFR mutation-type advanced NSCLC. The 5-year survival rate of patients with EGFR-mutant metastatic lung adenocarcinoma treated with EGFR-TKIs is 14.6%.^[2]^ Patients with advanced NSCLC with long-term survival (>10 years) are still rare. This is the first known report of a patient with advanced NSCLC, advanced non-smoking lung adenocarcinoma, and no EGFR mutation who survived for nearly 12 years by comprehensive treatment based on EGFR-TKI-based therapy.

## 2 Case presentation

**Figure 1:**
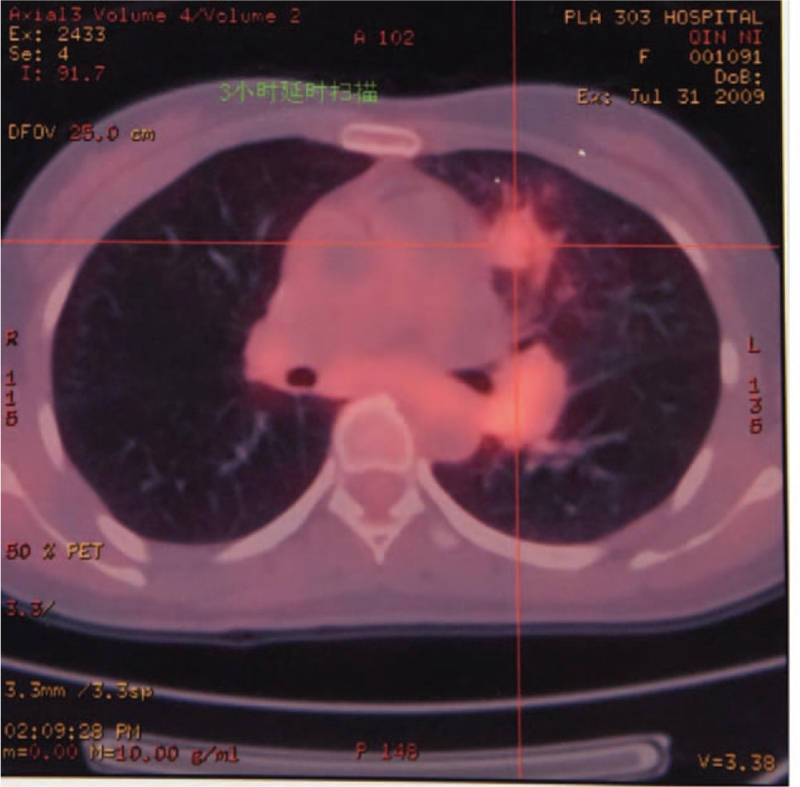
The whole body F18-FDG PET/CT tomography scan (July 31, 2009) showing a pulmonary nodule on upper lobe of the left lung. The radioactive intake is moderate and a nodule on left Lung Gate. F18-FDG-PET/CT = Fluorine 18 fluorodeoxyglucose- Positron emission tomography/computed tomography.

**Figure 2:**
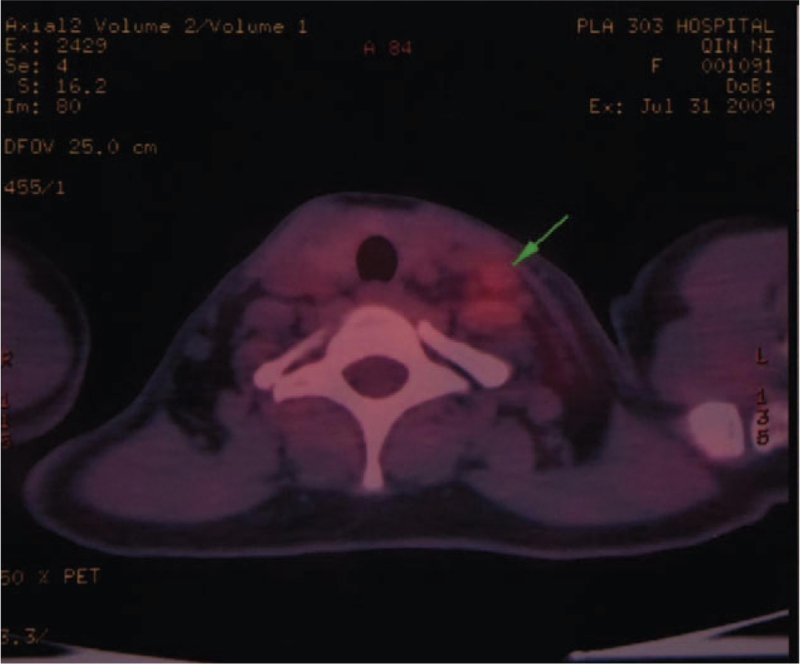
The whole body F18-FDG PET/CT tomography scan (July 31, 2009) showing multiple enlarged lymph nodes on left supraclavicular area. F18-FDG-PET/CT = Fluorine 18 fluorodeoxyglucose- Positron emission tomography/computed tomography.

A 48-year-old Chinese woman was admitted to our hospital on July 25, 2009, because of a left supraclavicular mass that had persisted for 2 months. The patient's left supraclavicular mass was painless. The patient had no clinical symptoms, such as cough, anhelation, hoarseness, chest pain, dysphagia, upper abdominal swelling, and no history of smoking or family history of tumors. Physical examination revealed that multiple enlarged lymph nodes were touched on left supraclavicular mass, their diameters in size were 0.8 to 1.4 cm. It has the characteristics of a hard texture, fusion, and unclear boundaries. The skin temperatures of the local masses were normal. The lymph node was not palpable in the right supraclavicular region. The whole body F18-fluorodeoxyglucose-positron emission tomography/computed tomography (July 31, 2009) revealed that there was a pulmonary nodule on upper lobe of the left lung. The boundary of the nodule was unclear with spiculation sign, and the size was about 1.5 cm × 1.4 cm. The radioactive intake was moderate. There was a nodule with radioactive intake with mild concentration on left lung, and the size was about 2.7 cm × 2.4 cm (Fig. 1). No placental lesions or radioactive intake abnormalities were found bilaterally in the breasts. Multiple enlarged lymph nodes with highly radioactive ingestion were found in the left supraclavicular area, with diameters in size were 0.4 to 1.4 cm (Fig. 2). Nasopharyngeal morphology was normal, and there were no radioactive abnormalities. There were no masses in the abdomen or abdominal wall. Diagnosis by gastroscopy (July 30, 2009) showed chronic inflammation of chronic erosive gastritis and chronic inflammation of the duodenum, and the esophagus was not found to be abnormal. Laboratory findings were within the normal range, except for the cancer antigen 125 (CA125) level of 104.4 U/mL (normal range is 0–35.00 U/mL) in the serum. We performed a B-type ultrasonography-guided core needle biopsy of the left supraclavicular lymph nodes, and the pathological diagnosis of the specimen was metastatic adenocarcinoma (July 31, 2009). The final diagnosis was left lung adenocarcinoma with left supraclavicular lymph node metastasis, cT1N3M0 IIIB (International association for the Study of lung cancer 6th version). The patient was treated with chemotherapy including gemcitabine (1.6 g/m^2^ d1, d8) and cisplatin (40 mg/m^2^, d1–3) for 2 cycles: gemcitabine (1.6 g/m^2^ d1, d8) and carboplatin (area under the curve [AUC] = 5, d1), for 2 cycles between August 5, 2009, and November 17, 2009. Since then, the patient rejected the above-mentioned chemotherapy regimen and switched to EGFR-TKI targeted therapy. However, the application of targeted therapy with EGFR-TKIs has not been advocated for patients with advanced NSCLC harboring wild-type EGFR. Although gene mutations in patients’ lesions have been detected, it was reported that the clinical features of adenocarcinoma in female non-smokers positively correlated with the effects of EGFR-TKI drugs. Starting on January 15, 2010, we prescribed gefitinib to this patient at a daily dose of 0.25 for maintenance treatment. The patient had no obvious adverse reactions during the course of the therapy. As of March 1, 2010, CT imaging of the patient's chest showed that the left supraventricular lymph nodes and left lung lesions had become smaller (CA125: 38.9 U/mL). The efficacy evaluation was partial response (PR) (response evaluation criteria in solid tumors). The patient recovered very well and continued to use targeted therapy with gefitinib alone at home during this period. On July 6, 2015, the patient returned to the hospital because of a headache. Magnetic resonance (MR) imaging of the brain (July 9, 2015) showed thickening of the meningeal and arachnoid regions and suspected metastatic lesions. MR imaging of the chest (July 9, 2015) showed that the clavicle and pulmonary lesion had disappeared, CA125 level was 15.6 U/mL. We performed local radiotherapy (40 GY/20 F) in patients with brain metastatic lesions from on July 20 to August 25, 2015. After radiotherapy, the patient's headache symptoms improved. Since September 2015, 80 mg of osimertinib has been administered daily as maintenance therapy at home, with no abnormal changes in her body and no obvious side effects. By on May 12, 2020, the patient experienced weakness in the lower limb myodynamia but had no obvious headaches, coughs, or other symptoms. The results of the driver gene detection for EGFR, anaplastic lymphoma kinase (ALK), and C-ros oncogene1 receptor-tyrosine kinase (ROS1) in cancer cells from peripheral blood samples were negative (July 21, 2020). Despite this, considering that patients had received osimertinib for >5 years, we continued to administer double-dose osimertinib (160 mg, quaque die Per os [PO]) alone, but the muscle strength of the lower limbs did not improve significantly. On August 15, 2020, neurological examination was performed, and the muscle strength of the lower limbs was grade 2. MR imaging (August 26, 2020) showed thickening of lumbar arachnoid membrane and suspected metastatic lesion. Chest CT scan (August 25, 2020) showed that there was a metastatic lesion in the lungs with a size of 1.7 cm × 3.3 cm (progressive lesion), and no lesions were seen on the left supraclavicular. CA125 levels were 60.68 U/mL. There were no inflammatory or cancer cells in the cerebro spinal fluid from the lumbar puncture. We considered that the lung lesions acquired resistance to osimertinib. From September 8, 2020, to November 21, 2020, the patient was administered combination chemotherapy pemetrexed (0.7 g, d1) with oxaliplatin (195 mg, d1). Meanwhile, the lumbar lesion was treated with hyperthermia, 40° for 1 hour, d1–4, for 4 cycles. By December 16, 2020, a chest CT scan showed progression of the lung lesion. The lumbar arachnoid and chorionic membranes were pinched. His muscle strength improved to grade 4. From December 20, 2020, we decided to combine toripalimab (240 mg, d1) with anlotinib (12 mg, quaque die, for 2 weeks, 1 week rest), q3w for 4 cycles. By April 22, 2021, physical examination revealed that the symptoms of muscle strength had recovered to Grade 4. MR imaging revealed that the lumbar arachnoid and chorionic membranes were not thickened (April 27, 2021). Chest computed tomography (CT) scan (April 23, 2021) showed enlargement of the lung metastatic lesion (3.1 cm × 4.1 cm). Efficacy evaluation was based on the progressive disease (response evaluation criteria in solid tumors criteria). The results of the driver gene detection for EGFR, ALK, and ROS1 in cancer cells from peripheral blood samples were negative (May 15, 2021). Although the genetic test results were negative, the patient had always benefited from treatment with EGFR-TKi drugs since 2010 and did not benefit from combined immunity with antiangiogenic therapy. Double-dose icotinib treatment can benefit patients with previous resistance to EGFR-TKIs. Therefore, from May 20, 2021, the patient self-administered double-dose icotinib (125 mg × 2, Ter in die Per os [PO]) alone (the whole treatment process is shown in Table 1). However, on June 19, 2021, the patient suddenly appeared comatose. Chest computed tomography (CT) (June 19, 2021) showed that the lung metastatic lesion had shrunk (1.7 cm × 1.8 cm, and original size: 3.1 cm × 4.1 cm). Efficacy evaluation was performed using the PR (response evaluation criteria in solid tumors criteria). Unfortunately, MRI of the brain showed brain infarction in multiple parts of the corona radiata, frontal lobe, and left cerebellar hemispheres (June 19, 2021). Finally, the patient died of brain infarction on July 23, 2021. The patient has been alive for nearly 12 years (July 25, 2009--July 23, 2021) with comprehensive EGFR-TKI-based therapy.

**Table 1:**
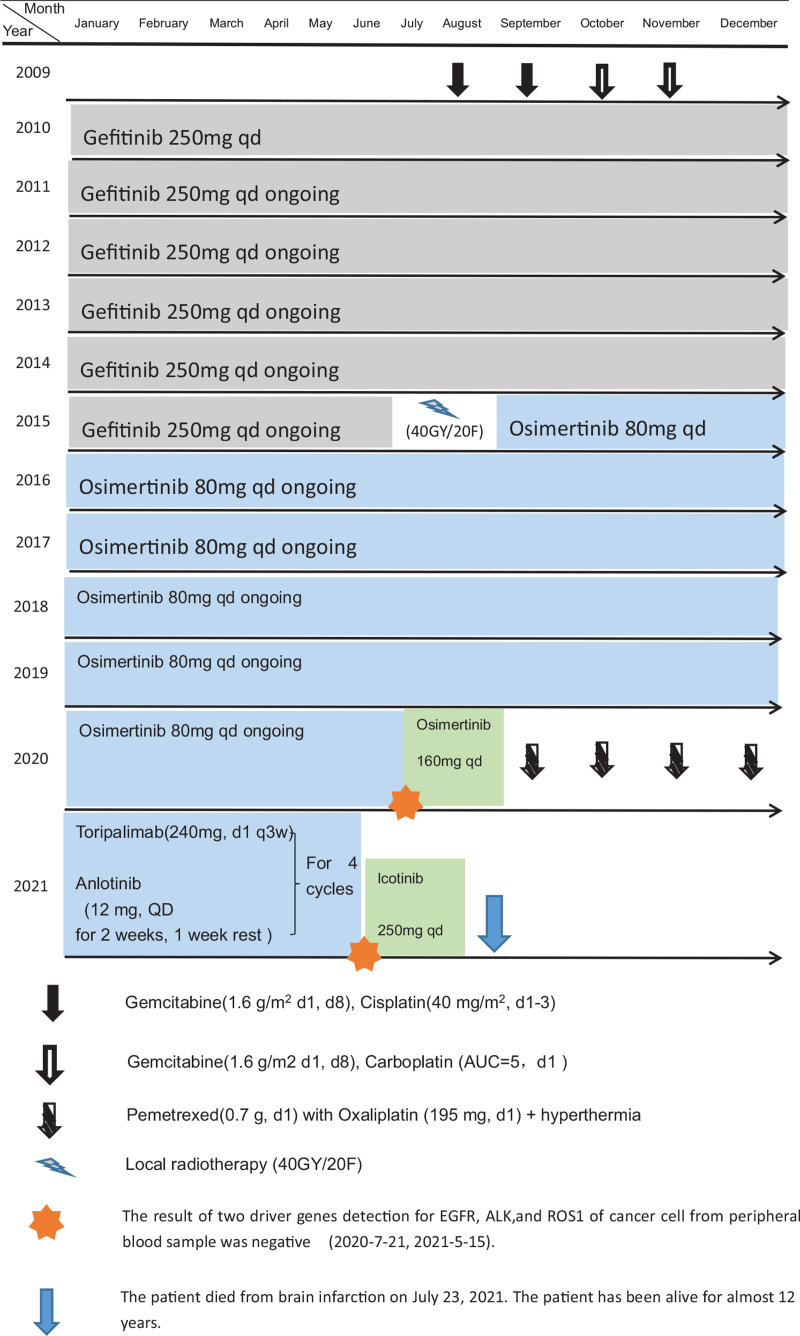
Treatment course for patient in 2009 to 2021.

## 3 Discussion

Detection of sensitive mutations in genes, such as EGFR, ALK, and ROS1, is required for advanced non-squamous cell carcinoma of NSCLC. If the EGFR gene is mutated, we can use first- and third-generation EGFR-TKI treatments. Reports showed that the First-generation targeted drugs such as gefitinib, icotinib, and erlotinib have better progression-free survival (PFS) than chemotherapeutic drugs. Maintenance therapy with gefitinib after induction chemotherapy may improve the OS of patients with NSCLC. Research^[3]^ showed that the PFS of maintenance therapy group and recurrent group after induction chemotherapy was 16.5 months (95% CI: 8.7–24.3) and 9.2 months (95% CI: 7.5–10.9), respectively, and the difference between the 2 groups is statistically significant (*P* = .0000). In addition, the median MS in the maintenance therapy group was significantly correlated with the smoking status, pathology type, liver metastasis, and objective response to gefitinib. The patient we reported was one with 2 driver genes for EGFR; however, it was reported that some clinical features such as Asian, woman, non-smoker, and adenocarcinoma positively correlated with the efficacy of EGFR-TKI drugs. Gefitinib is more effective in patients with adenocarcinoma, women, and non-smokers with NSCLC.^[4]^ Therefore, the patient in this report also received targeted maintenance therapy after chemotherapy with gefitinib alone. The patient's PFS was 5.5 years, which is much longer than that reported in the literature.^[3]^

Osimertinib, a third-generation EGFR tyrosine kinase inhibitor, can efficiently penetrate the blood-brain barrier. It is a promising treatment option for EGFR-mutated NSCLC with LM, regardless of T790M mutation status. Osimertinib has been reported to have a very good clinical effect in patients with NSCLC of T790M positive after resistance to gefitinib. The patient in this report developed meningeal and arachnoid metastatic lesions in the brain after receiving gefitinib therapy; therefore, we considered that the patient was resistant to gefitinib. The patient had headache symptoms when the gene mutation status was unknown and underwent whole-brain radiotherapy. After radiotherapy, osimertinib alone was used as maintenance therapy for 4.5 years. Patients with 19del/L858R of NSCLC by osimertinib gained ideal positive results after resection of EGFR-mutated non-small-cell lung cancer, and reduced the risk of recurrence and death, such as disease-free (overall hazard ratio for disease recurrence or death: 0.17; 95% CI [0.11–0.26]; *P* < .001), and improved DFS.^[5]^ The patient we reported had advanced NSCLC and was treated with osimertinib. The PFS was much longer than that reported in other literature.^[6]^

Double-dose Icotinib, as compared with routine-dose icotinib (125 mg, thrice daily), significantly prolonged the median PFS (mPFS) in patients with exon 21 L858R mutation from 9.2 to 12.9 months, and increased the ORR rate.^[7]^ Although the genetic test results were negative, the patient we reported had always benefited from treatment with EGFR-TKi drugs since 2010, and did not benefit from combined immunity with antiangiogenic therapy. The patient was self-administered double-dose icotinib (125 mg × 2, Ter in die Per os [PO]) alone. After 1 month, the efficacy evaluation was PR for lung metastatic lesions. This is consistent with a previous literature report.

In conclusion, our case is of significant importance to clinicians involved in the treatment of patients with advanced non-smoking lung adenocarcinoma and no EGFR mutations.

## Author contributions

Qianyu Zhang: Study conceptualization and original draft writing. Meiling Liu: Study conceptualization and original draft writing. Yunchao Zhong: Original draft writing. Kang Zhang: Study conceptualization, manuscript writing, reviewing and editing, study supervision, and project administration. All authors have read and approved the final manuscript.

**Conceptualization:** Kang Zhang, Qianyu Zhang.

**Resources:** Meiling Liu, Yunchao Zhong.

**Visualization:** Kang Zhang.

**Writing – original draft:** Qianyu Zhang.

**Writing – review & editing:** Kang Zhang, Qianyu Zhang.
